# Accounting for Bacterial Overlap Between Raw Water Communities and Contaminating Sources Improves the Accuracy of Signature-Based Microbial Source Tracking

**DOI:** 10.3389/fmicb.2018.02364

**Published:** 2018-10-02

**Authors:** Moa Hägglund, Stina Bäckman, Anna Macellaro, Petter Lindgren, Emmy Borgmästars, Karin Jacobsson, Rikard Dryselius, Per Stenberg, Andreas Sjödin, Mats Forsman, Jon Ahlinder

**Affiliations:** ^1^Division of CBRN Security and Defence, FOI, Swedish Defence Research Agency, Umeå, Sweden; ^2^Surgery Section, Department of Surgical and Perioperative Sciences, Umeå University, Umeå, Sweden; ^3^National Food Agency, Uppsala, Sweden; ^4^Department of Ecology and Environmental Science (EMG), Umeå University, Umeå, Sweden; ^5^Computational Life Science Cluster (CLiC), Department of Chemistry, Umeå University, Umeå, Sweden

**Keywords:** microbial source tracking, fecal contamination, bacterial community analysis, microbial community profiling, 16S rRNA amplicon

## Abstract

Microbial source tracking (MST) analysis is essential to identifying and mitigating the fecal pollution of water resources. The signature-based MST method uses a library of sequences to identify contaminants based on operational taxonomic units (OTUs) that are unique to a certain source. However, no clear guidelines for how to incorporate OTU overlap or natural variation in the raw water bacterial community into MST analyses exist. We investigated how the inclusion of bacterial overlap between sources in the library affects source prediction accuracy. To achieve this, large-scale sampling – including feces from seven species, raw sewage, and raw water samples from water treatment plants – was followed by 16S rRNA amplicon sequencing. The MST library was defined using three settings: (i) no raw water communities represented; (ii) raw water communities selected through clustering analysis; and (iii) local water communities collected across consecutive years. The results suggest that incorporating either the local background or representative bacterial composition improves MST analyses, as the results were positively correlated to measured levels of fecal indicator bacteria and the accuracy at which OTUs were assigned to the correct contamination source increased fourfold. Using the proportion of OTUs with high source origin probability, underpinning a contaminating signal, is a solid foundation in a framework for further deciphering and comparing contaminating signals derived in signature-based MST approaches. In conclusion, incorporating background bacterial composition of water in MST can improve mitigation efforts for minimizing the spread of pathogenic and antibiotic resistant bacteria into essential freshwater resources.

## Introduction

Access to clean water is of global importance, and so critical to human wellbeing that it was identified as the main risk to society (World Economic Forum, 2015). Presently, worldwide health is being challenged by the spread of pathogens and antibiotic-resistant bacteria through the fecal pollution of freshwater. The diversity of fecal sources, e.g., urban wastewater and stormwater release, private sewage, animal farming and wildlife, has caused chronic freshwater pollution in many locations. This adverse impact on water quality is expected to be magnified by population growth and climate change (Vörösmarty et al., 2000). As such, the evaluation of water quality, identification of possible sources of pollution and decontamination of those sources are important steps to preventing the spread of waterborne diseases.

Fecal pollution of water bodies can also occur as episodic contaminations from point sources. In these cases, microbial source tracking (MST) works to determine the sources of the introduced fecal bacteria. However, the chronic release of fecal material at a subtle, but persistent rate can create mixed signatures between the environmental water and fecal sources without necessarily elevating the abundance of classic fecal indicator bacteria (FIB). Long-term anthropogenic effects, which have globally affected water sources and markedly decreased general water quality (Evans et al., 2005; Lotze et al., 2006; Halpern et al., 2008), are most likely accompanied by increased levels of fecal-related bacteria. In addition, as many bacteria demonstrate a cosmopolitan distribution, they can occupy different niches and occur in both water and fecal environments (McLellan and Eren, 2014). These potential overlaps should be considered in a MST analysis to avoid false-positive results in terms of overestimations of the amount of fecal material in the water and erroneously identified sources, both of which could mislead mitigation efforts.

High-throughput sequencing techniques are rapidly becoming the gold standard in microbial community analyses. One such technique is 16S ribosomal RNA (rRNA) amplicon sequencing. Amplicon rRNA-sequencing is a culture-free method that is noticeably faster than traditional culture-based methods and enables researchers to analyze the entire microbial community within a sample. Furthermore, the method is cost-effective, as many samples can be combined in a sequencing run. In light of MST, 16S rRNA amplicon sequencing has mostly been used to identify new fecal pollution indicators and target different bacterial orders within the dominant fecal phyla Bacteroidetes and Firmicutes, such as Bacteroidaceae, Clostridiaceae, and Lachnospiraceae (McLellan and Eren, 2014). As an alternative, operational taxonomic unit (OTU) abundance combined with statistical classification methods can determine the proportions of potential pollution sources in a MST sample (i.e., a sink community) (Smith et al., 2010; Knights et al., 2011; Casanovas-Massana et al., 2015). This approach requires a library of microbial communities that are representative of source environments believed to be contaminating the local environment (Ahmed et al., 2015; Dubinsky et al., 2016; Brown et al., 2017). One popular signature-based approach, the SourceTracker software (Knights et al., 2011), leverages a Dirichlet-Multinomial model to infer pathogen proportions in the sink community.

There is no consensus for how to define the reference library in the signature-based MST approach; for example, should the natural water community be present in the source tracking library as a proxy for background bacterial composition? This leads to another question: if no such representative background communities are available, what can be done instead? Some recent studies (Newton et al., 2013; Ahmed et al., 2015, 2017; Brown et al., 2017) have used locally sampled fecal sources, yet no samples describing the natural variation in bacterial community composition of sink samples were included in their source tracking libraries. When studying the impact of human-associated fecal material at recreational coastal sites of Australia (Victoria), Henry et al. (2016) included bay and river water communities in the library together with likely fecal and human-associated sources of contamination. Dubinsky et al. (2016) performed signature-based MST along swimming beaches of the Russian River watershed in California. They used a library of human waste, animal fecal communities, and blank samples to account for contamination during analytical procedures, and background water samples with low FIB counts from the same watershed to control for the influence of local, prevalent microbial communities. In this way, there is strong variation in how sequence-based, library-dependent MST approaches have been carried out with respect to the inclusion of background water communities, and an evaluation of alternative library definitions would be of interest to the MST community.

Most studies using signature-based MST to assess water quality have focused on the prediction of contaminations (e.g., Ahmed et al., 2015, 2017). Only limited research has assessed the taxonomic composition and signal strength in contaminated water samples. These approaches could provide insight into how bacterial communities interact and evolve in aquatic environments. For example, even though the estimated proportion of a certain contaminant is large with small standard deviation, the signal might include many taxa that commonly occur in the local water and thus, represents a false-positive. Brown et al. (2017) assessed the content of goose fecal matter and treated wastewater effluent contaminations at Lake Superior, Minnesota by investigating the abundance of the most common taxa underpinning each signal: the reported taxa was identified as well-known fecal community members in the literature. Further efforts in this direction would provide evidence for the reliability of the signature-based method and information about how common fecal sources affect the microbiome when released into water.

This study aimed to evaluate how accounting for bacterial overlap in the MST library affects source tracking accuracy. A large-scale sampling effort yielded 397 fecal (including feces from eight different host species) and background raw water samples (collected between 2013 and 2015 from six different locations), which were then sequenced and analyzed. We improved prediction of the correct fecal source by accounting for the bacterial overlap that exists in contaminating sources and local water.

## Materials and Methods

### Water Sample Collection

**FIGURE 1:**
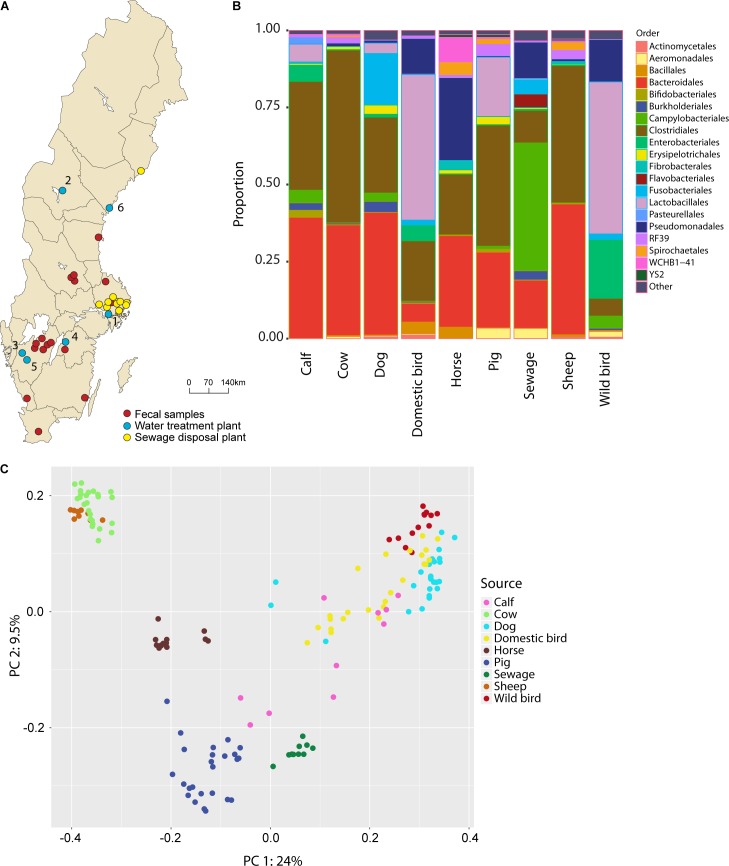
**(A)** Map of sampling sites, including raw water, feces, and sewage inlets. The water treatment plants were located in: (1) Stockholm; (2) Östersund; (3) Trollhättan; (4) Motala; (5) Borås; and (6) Härnösand. **(B)** Taxonomic composition, at the order level, of the contaminating sources included in the MST library. All communities in each source environment are concatenated. **(C)** PCoA plot showing transformed distances between rarefied fecal library assemblages using unweighted UniFrac distances.

Raw water samples were collected from inlets at six different water treatment plants in Sweden (**Figure 1A** and **Table 1**). A total of 175 raw water samples were collected between September 2013 and February 2015. These water treatment plants were selected from a large number of candidates because of fundamentally different traits, i.e., FIB levels (*E. coli* and coliforms), chemical oxygen demand and turbidity. Sixty liters of raw surface water were concentrated using dead-end ultrafiltration with Rexeed 25AX filters (Asahi Kasei Corporation, Tokyo, Japan) at a filtration rate of 2 L per minute (Smith and Hill, 2009) and eluted for a final volume of 600–700 mL. The filters had been pretreated with fetal calf serum (PAA Laboratories, Waltham, MA, United States) to prevent the adhesion of microorganisms. Filters were then returned to the National Food Agency (Uppsala, Sweden), where concentrates were eluted using back-flushing with 500 mL elution buffer [phosphate-buffered saline containing 1% Tween 80 and 0.01% Antifoam A (both from Sigma-Aldrich, St. Louis, MO, United States)] for a final volume of 600–700 mL. *E. coli*, coliforms and enterococci were detected from unconcentrated water that was collected in parallel using Colilert^®^ and Enterolert^®^-E kits according to the manufacturer’s instructions (IDEXX, Hoofddorp, Netherlands). For DNA isolation, 2 ml of water was centrifuged at 16,000 ×*g* for 1 h, after which 1.9 mL of the resulting supernatant was discarded and DNA was extracted from the remaining volume using a SoilMaster DNA Extraction Kit according to the manufacturer’s recommendations for environmental water samples (Epicentre Biotechnologies, Madison, WI, United States). To increase DNA yield, the samples were treated with proteinase K and incubated at 37°C for 10 min without shaking. The resulting DNA pellet was resuspended in 60 μL of TE buffer and either frozen and stored or immediately subjected to PCR analysis. Sample preparation, PCR reaction preparation and thermal cycling were performed in separate rooms.

**Table 1:** Statistics regarding fecal indicator bacteria (FIB) levels and phylogenetic diversity (PD; Faith and Baker, 2006) in the samples from raw water sources, collected between 2013 and 2015.

	Water source	*Coliforms* (MPN/100ml)	*E. coli* (MPN/100ml)	*Enterococci* (MPN/100ml)	Phylogenetic diversity
Stockholm	Major lake	69.6 (183.9)	1.6 (1.7)	1.07 (2.02)	96.3 (25.1)
Östersund	Major lake	10.4 (27.8)	3.1 (10.1)	0.26 (0.53)	74.8 (13.6)
Motala	Major lake	58.9 (164.2)	0.36 (0.90)	0.36 (0.72)	77.1 (24.1)
Borås	Minor lake	155.9 (413.7)	15.6 (12.4)	5.13 (7.99)	87.5 (14.0)
Härnösand	Minor lake	61.6 (145.2)	0.55 (1.38)	0.86 (1.73)	79.8 (17.6)
Trollhättan	Major river	337.2 (697.3)	89.3 (199.5)	29.7 (92.7)	92.4 (19.2)

*Average values are shown with standard deviation in parentheses.*

### Fecal and Sewage Reference Sample Collection

A total of 212 fecal samples from seven species of wild and domestic animals that are likely to pollute raw water in Sweden were collected between autumn 2013 and spring 2015. The collected feces included calf, cow, dog, domestic bird, horse, pig, sheep, and wild bird feces. To obtain a wide geographical spread for the samples, official veterinarians from several regions across Sweden were involved in the sample collection (**Figure 1A** and **Table 1**). Samples were transported on ice and analyses were started within 24 h of collection. DNA preparation was performed using a QIAamp DNA Stool Mini Kit (Qiagen) following the manufacturer’s instructions. A total of 10 untreated sewage samples were collected from eight different municipal and private wastewater treatment plants (**Supplementary Table 1**), with 50 ml of unprocessed sewage collected at the inlet and transported on ice. DNA from the untreated sewage samples was isolated using the SoilMaster DNA Extraction Kit as described above.

### Amplicon Preparation and Sequencing

DNA was amplified using the No. 5 Hot Mastermix 2.5x kit (5 PRIME, Hilden, Germany) with bacteria/archaeal primers 515F/806R specific for the hypervariable V4 region of the 16S rRNA gene (Caporaso et al., 2012). The forward and reverse primers were modified to incorporate a 12 bp Golay error-correcting barcode that enables sample multiplexing (Caporaso et al., 2012). All samples were amplified in triplets and pooled after PCR amplification (94°C for 3 min; 35 cycles of 94°C for 45 s, 50°C for 1 min, 72°C for 1.5 min; 10 min rest to finish). The PCR product was run on a 1% agarose gel and the DNA concentration was estimated with a Qubit fluorometer (Invitrogen, Carlsbad, CA, United States). The amplicons were pooled at equimolar concentrations and purified with the Ultra Clean PCR Clean-Up kit (MoBio, Carlsbad, CA, United States) following the supplier’s instructions. The DNA concentration of the pooled amplicon product was measured with a Qubit fluorometer and adjusted to 2 nM. The library was denaturated and diluted as described by Illumina (MiSeq System User Guide, Part # 15027617 Rev. C), before it was loaded onto a MiSeq cartridge (Illumina, San Diego, CA, United States) and sequenced using a 500 bp paired-end sequencing protocol.

### Quality Control and Raw Data Processing

The Quantitative Insights Into Microbial Ecology (QIIME) pipeline, version 1.9, was used for processing raw sequence data (Caporaso et al., 2010). After demultiplexing and quality filtering using default parameter values, the remaining adapter sequences were removed using cutadapt, version 1.2.1 (Martin, 2011). Associated forward and reverse reads were merged using FLASH (Magoč and Salzberg, 2011). Chimeric reads were detected and removed from the data using USEARCH, version 6.1 (Edgar, 2010). The obtained reads were then matched to the reference database Greengenes, version 13.8, based on 97% sequence identity (McDonald et al., 2012) and clustered into OTUs through a closed-reference approach using UCLUST (Edgar, 2010), with taxonomies assigned to the representative sequence of each OTU.

Throughout the study, low abundance OTUs were filtered out from the data following recommendations by Bokulich et al. (2013) on Illumina-generated amplicon data: singletons and OTUs with less than 0.001% of the total number of reads were removed. In addition, OTUs were retained only if they were observed in a minimum of three samples. Alpha diversities were estimated using the phylogenetic diversity metric (PD whole tree; Faith and Baker, 2006). A principal coordinate analysis (PCoA) was performed based on UniFrac distance (Lozupone and Knight, 2005) of the rarefied sequence data, where the rarefaction level was set to 15,000 sequences. Taxa composition and PCoA were visualized using the R package phyloseq (McMurdie and Holmes, 2013). The number of OTUs shared between environments was calculated using the script shared_phylotypes.py in QIIME after rarefaction to a depth of 1,000,000 sequences per source environment.

### Selection of Background Microbiomes

**FIGURE 2:**
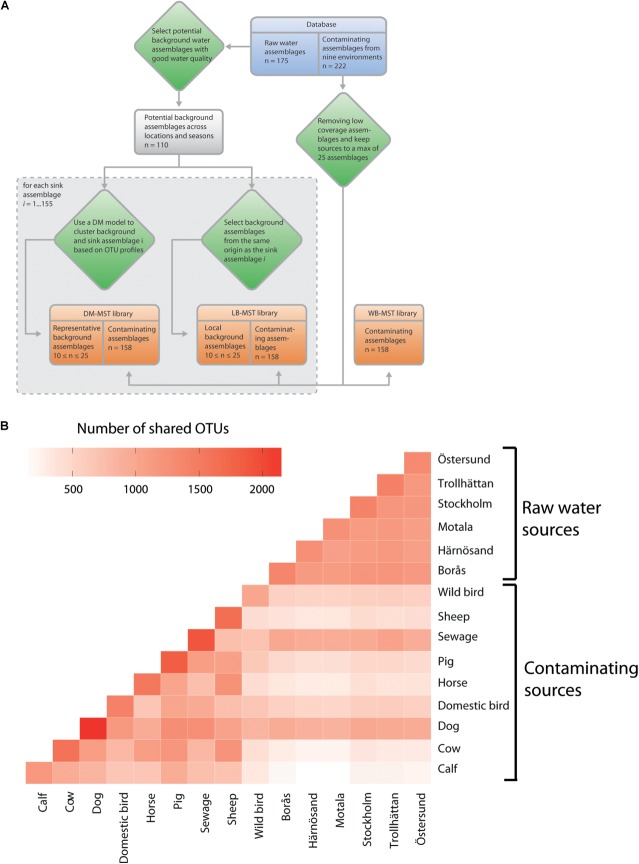
**(A)** Overview of how the three microbial source tracking (MST) library setups for source tracking analyses incorporate raw water communities, with the 155 sink assemblages representing a subset of the total group of 175 raw water assemblages. **(B)** Shared operational taxonomic units (OTUs) between different contamination sources and the six sampled raw water sites. Each source of pooled samples was rarefied to an even depth of 1,000,000 reads. Source environments are ordered in contaminating and raw water sources.

Three alternatives were considered when establishing the MST library for each sink sample (**Figure 2A**). The first option, referred to as the “without background” representative library (WB-MST), was a library that only represented fecal and sewage sources, i.e., background water communities were excluded.

The raw water samples that were classified as potential background samples and included in the cluster analysis were classified as uncontaminated according to FIB levels (*E. coli* <25 MPN/100 ml and coliforms <100 MPN/100ml). A preliminary MST analysis included all the water communities from the same location that satisfied the background FIB levels. Communities which showed fecal contamination were believed to have been contaminated during sample processing and were thus excluded from the group of potential background samples (**Supplementary Material 1**). A model-based clustering analysis (Holmes et al., 2012) of the selected potential background samples and the sink samples was performed in the R package DirichletMultinomial (Morgan, 2014). Between two and eight Dirichlet components were fitted to the data so that the optimal number of components could be identified. The number of components that yielded the best model selection score based on the Laplace approximation criterion was chosen. The number of samples selected to represent background water corresponded to the number of samples representing sources of contamination in the library. For each sink sample, the water communities that clustered together were selected as representative background communities in the source tracking library, after which an optimal partition, including between 10 and 25 communities in the same group as the sink sample, was chosen. If the desired number of communities was not met, the second most optimal partition was chosen and so forth until a reasonable number of communities was selected. This approach is referred to as Dirichlet Multinomial selected background representative library MST (DM-MST).

To build a library which included locally representative, good-quality water communities, we removed atypical samples that did not meet the FIB score criteria described above. A preliminary MST analysis was then performed, and samples with less than 0.99 estimated assignment probability to the background water source were not considered as a background representative community. This approach is referred to as the local background representative library MST (LB-MST).

### Source Tracking Analyses

SourceTracker, version 1.0.0 (Knights et al., 2011), was utilized for performing the MST analyses on 155 out of the 175 raw water samples, after removing samples contaminated during lab processing, delayed during delivery and sampled less than 48 h apart (see **Supplementary Material 1**). The training set employed a rarefaction depth of 10,000 sequences following recommendations by Ahmed et al. (2015). Sink samples were not rarefied to maximize the taxa resolution and facilitate the identification of low abundance OTUs of possible fecal origin. Default values were used for all the other parameters in SourceTracker based on suggestions by Henry et al. (2016). To calculate the posterior probability of an OTU belonging to a certain source environment for multiple sink samples, we modified the script sourcetracker_for_qiime.R provided in the SourceTracker package by normalizing the posterior probability of each OTU to one for each sink sample. The modified function is provided at http://github.com/FOI-Bioinformatics/MST. The method requires a library of potential contaminating sources. To be included in the library, samples had to consist of more than 15,000 reads, which corresponds to a lower limit, and each source consisted of 10–25 samples. For those environments with more than 25 communities available, one or a few of the communities sampled from the same geographic location were removed (**Supplementary Material 1**). A leave-one-out cross-validation (LOO-CV) analysis of the library, implemented in the SourceTracker software, was carried out to investigate the ability to discriminate between potential contaminating sources.

The signals were visualized using the graphlan package (Asnicar et al., 2015), and OTUs with estimated posterior probabilities of source assignment greater than 0.7 are highlighted in red. To avoid the inclusion of too many low probability OTUs, only OTUs with source probabilities greater than 0.1 were kept. The alluvial diagram was made using the online tool http://raw.densitydesign.org/, with each OTU assigned to its most probable source. A few OTUs with equal source probability were removed as no single source origin could be assigned.

### Statistical Analyses

A Wilcoxon rank sum test with continuity correction, implemented in the R statistical package (R Core Team, 2016), was used to determine whether between-group differences in mean values in source contaminating proportion were statistically significant. The contamination proportion for each community was obtained through MST analyses using three different library setups as described earlier.

Multivariate homogeneity of group dispersions was calculated for each environment to examine the variance of source environments in the library. This analysis was performed with the betadisper function in the R package vegan (Oksanen et al., 2013); Bray–Curtis dissimilarity index distances between individual communities and group centroids were handled by reducing the original distances to principal coordinates.

To estimate correlation parameters across water quality measures (i.e., estimated contaminations obtained from the MST analyses and FIB levels), a multivariate generalized linear mixed model (GLMM) was fitted to the data. The mean proportion of the response j for sample i could be assumed to be:

(1) $$\text{g}({\text{m}}_{ij})={\text{β}}_{jk}+{\text{u}}_{ij},$$

where g(.) is the link function, β*_jk_* is the location-level predictor variable (with numeric code *k*) where the sample *i* was taken for response *j*, *i* = 1,...,155, *k* = 1,...,6, and *j* = 1,...,6, and u*_ij_* is the residual of sample *i* for response *j*. Here, **u***_i_* = (u*_i1_*,...,u*_i6_*) is multivariate, normally distributed with a zero mean vector and covariance matrix with unknown parameters, which accounts for correlation between water quality measures. The log link function was used for count response data (i.e., for FIB data) while the identity link was used for contaminations. For each sample, all contaminating source proportions were added to a single score. These contaminations were log-transformed prior to analysis to satisfy parametric assumptions of normality. FIB levels lower than one unit per 100 mL were assumed to be zero. To avoid inducing strong auto-correlations, communities sampled less than 48 h apart were removed (three samples). Eqn. (1) corresponds to the first level of the hierarchical model, while the location-specific regression is the second level of the model:

(2) $${\text{β}}_{jk}={\text{β}}_{j}+{\text{u}}_{jk}^{},$$

where β*_j_* is a vector of response-specific coefficient and u*_jk_* is the error term of location *k* for response *j*. The inclusion of location-level predictor variables ensured that correlation between locations was taken into consideration.

To detect differences in the source accuracy of the obtained fecal signals between WB-MST, DM-MST, and LB-MST analyses, the OTUs with posterior probability of source assignment greater than 0.7 were analyzed. A GLMM with a log link function and total number of OTUs as an offset was used to analyze the data. Contaminants with low estimated proportions (less than 0.3%), which may nevertheless exist within the noise threshold, were not considered so that unrepresentative proportions of OTUs could be avoided. In addition, contaminating signals from communities sampled less than 48 h apart were removed. The mean number of OTUs behind each signal could be written as:

(3) $$\text{g}({\text{m}}_{l})={\text{a}}_{l}+{\text{β}}_{ik}+{\text{β}}_{lm}+{\text{β}}_{1}{\text{x}}_{1l}+{\text{β}}_{2}{\text{x}}_{2l},$$

where a*_l_* adjusts for the total number of OTUs underlying signal *l* (*l* = 1,...,496), β*_ik_* is a predictor for a sample *i* within location *k*, β*_lm_* is a source-specific coefficient (*m* = 1,..., 11) behind signal *l*, and β_1_ and β_2_ are regression coefficients related to whether a DM selected background (x*_1l_* = 1, x*_2l_* = 0) or a local background (x*_1l_* = 0, x*_2l_* = 1) was used for obtaining signal *l*. Level two of the model can be written as:

(4) $${\text{β}}_{ik}={\text{β}}_{k}+{\text{u}}_{ik},$$

where β*_k_* is a vector of location-specific coefficient, u*_ik_* is the residual for sample *i* within location *k*. The final level of the model can be expressed as:

(5) $${\text{β}}_{k}={\text{β}}_{0}+{\text{u}}_{k},$$

where β*_0_* is the overall intercept in the model and u*_k_* is the residual for location *k*. The models were implemented in the Bayesian software package rstan (Carpenter et al., 2017). In both regression analyses, Cauchy distributions with location parameter x_0_ = 0 and scale parameter γ = 5 were assigned to the standard deviation parameters while weakly informative normal distributions with mean μ = 0 and variance σ^2^ = 10 were assigned to the regression coefficients. In the first model (eqns. 1,2), a LKJ prior distribution, with shape parameter η = 1 (i.e., a uniform density) was assigned to the correlation matrix, and Cholesky factorization was used to speed up computations. In total, 8 parallel chains were run for 1,000 iterations each. Samples obtained in the first 500 iterations were discarded as burn-in.

## Results

### Screening of Raw Water Communities

Raw water samples, consecutively collected over a period of 18 month, from the inlets of six Swedish water treatment plants were sequenced (**Figure 1A** and **Table 1**). These water sources reflect diverse sources of contamination, human and agricultural activities, weather conditions, and water trophic and nutrient status. The hypervariable V4 region of the 16S rRNA gene was amplified and sequenced, after which community profiles were generated for all six locations from a total of 175 samples. Following quality filtering, 22 million reads were obtained. The reads were clustered into 1,616 OTUs at ≥97% similarity.

The overall quality of raw drinking water, in terms of FIB, varied greatly across locations. Trollhättan samples showed the highest average levels of *E. coli* and coliforms whereas the lowest levels were observed in Östersund, Motala, and Härnösand (**Table 1**). Phylogenetic diversity was greatest in raw water from Stockholm and Trollhättan, while Östersund raw water showed the least diversity. The most abundant orders were Actinomycetales (mean frequency 22.6%, minimum 1.1%, maximum 38.4% of the total number of reads), Burkholderiales (12.6%, 4.4–44.4%) and Flavobacteriales (8.3%, 0.2–60.3%) (**Supplementary Figure 3**). The abundance of some orders varied greatly between locations and seasons. For example, Alteromonadales was sometimes highly abundant in raw water from Stockholm (maximum 32.1%) and Motala (maximum 43.6%), and at other times not detected at all. Similarly, Methylococcales was detected in Östersund and Härnösand, but not detected or only found at low frequencies at other locations. Aeromonadales and Pseudomonadales were detected at all locations but the abundance of both orders varied between seasons, i.e., Aeromonadales was abundant during autumn in Borås (maximum 21.2%) and the abundance of Pseudomonadales increased during late summer and autumn in Trollhättan (maximum 33.5%).

### Differences in Community Composition Between Environments Enable Source Tracking Analysis

An extensive set of representative sources of contamination of raw water in Sweden was set up in order to perform MST analyses. Fecal samples from seven animal species, totaling 212 samples, were collected, from various locations (**Figure 1A** and **Supplementary Table 1**). In addition, ten untreated sewage influent samples were collected from eight wastewater treatment plants serving populations ranging from 500 to 171,000 people.

The taxa composition of the fecal sources, at the order level, was predominated by Bacteroidales, Lactobacillales, and Clostridales, which accounted for between 27% (sewage inlet) and 92% (cow feces) of the source environments (**Figure 1B** and **Supplementary Figure 1**). Some orders were only abundant in a few fecal groups: Fusobacteriales was present in dog feces (16.6%) and sewage (4.6%); Enterobacteriales was present in wild bird (18.9%), domestic bird (4.8%), and calf (5.2%) feces; and Verrucomicrobia was present in horse (8.0%) and sheep (1.6%) feces. Some striking similarities in taxa composition were observed between sources; notably, cow and sheep feces were dominated by Bacteroidales and Clostridiales (92 and 87%, respectively) while wild and domestic bird feces mainly consisted of Lactobacillales, Enterobacteriales, and Pseudomonadales (82 and 64%, respectively). No OTUs were present in all fecal communities. The most present OTU, assigned to the family Enterobacteriaceae, were detected in 90% of all samples. In addition, 4951 OTUs were present in only one sample, mostly in sewage environments (average *n* = 181 unique OTUs per sewage community).

To observe the differentiation across fecal bacterial communities, an ordination analysis was conducted. The first principal component (PC1) explained as much as 24.0% of the total community variation and separated cow and sheep fecal communities from dog and bird communities (**Figure 1C**). PC2 explained 9.5% of the variation and separated wild bird, cow, and sheep feces from pig feces and sewage. Among the fecal sources, dog, domestic and wild birds and calf displayed the largest within source variability, with an average Bray–Curtis distance to the centroid of 0.412, 0.438, 0.403, and 0.396, respectively. In contrast, sheep, sewage, cow and horse showed noticeably less within source variability, with an average distance to the centroid of 0.186, 0.240, 0.248, and 0.251, respectively. It was possible to trace each of the fecal communities to their true source in a LOO-CV analysis (**Supplementary Figure 2**), suggesting that each source group is well separable and can be further utilized in the MST analysis.

### OTU Overlaps Were Most Prominent Between Sewage, Dog Fecal and Raw Water Sources

After the raw water and contamination communities were characterized, the shared bacterial contents were investigated to better understand how the differences between alternative MST library setups might influence the results. OTU overlap was calculated once all the communities were merged according to their respective sources.

Not surprisingly, the raw water sources shared a large number of OTUs, with the samples containing between 1,080 and 1,200 shared OTUs (**Figure 2B**). The contaminating sources showed a more variable number of shared OTUs: a minimum of 343 OTUs were shared by wild bird and calf feces and a maximum of 1,282 OTUs were shared by dog feces and sewage sources. The highest and lowest average numbers of overlapping OTUs between raw water and all fecal sources were observed in the Stockholm (565.1, std dev of 259.4) and Motala water sources (479.1, std dev 266.8), respectively. Interestingly, sewage and dog fecal samples had the most OTUs in common with the raw water sources, ranging from 914 to 1,051 and from 870 to 993 shared OTUs, respectively. Other fecal sources showed a relatively small amount of OTU overlap with raw waters, e.g., calf samples (163 to 250 OTUs). We hypothesize that these differences in shared OTUs will negatively impact MST accuracy if they are not accounted for via representative water communities. The negative impacts could include bias in estimations of source proportions and increased false-positive results due to erroneously detected sources.

### Representative Water Communities Improve Correlation Between Estimated Contamination and FIB Abundance

To assess the performance of the signature-based MST methods with alternative library representations, we compared the contaminating proportions of sequence reads obtained in the MST analyses with traditional cultivation-derived water quality scores. The measured FIB scores were used as a reference for water quality score since this is an internationally recognized indicator of fecal contamination (World Health Organization, 2011). All of the estimated contaminating proportions obtained in the MST analyses were summed into a single score for each sink community, which served as a proxy for the total fecal load of the community. We expected that bias in estimated contaminating proportions would reduce correlations with FIB abundance.

Microbial source tracking analysis requires a library of bacterial profiles that are representative of the analyzed contaminating sources to classify the community. Here, three alternative library settings were investigated (**Figure 2A**). We refer to these settings as the local background representative library MST (LB-MST), Dirichlet Multinomial selected background representative library MST (DM-MST) and without background representative library MST (WB-MST) setups. Both of the setups that include background communities will identify signatures that deviate from natural variation in the taxonomic composition of the water shed, while the WB-MST setup will infer contaminations without any information about natural variation and thus report both chronic and temporary point sources of pollutions.

The WB-MST analysis found all of the raw water assemblages to be contaminated, with total contaminating proportions ranging from 0.001 to 0.760 and distributed mainly across dog, sewage and wild bird sources (**Supplementary Figure 4**). The LB-MST and DM-MST analyses (**Supplementary Figures 5**, **S6**) found 59 and 67 out of 155 communities, respectively, to be contaminated, with the criterion for contamination that the total proportion was greater than 0.3% and stemmed from a source represented in the library. These analyses suggested the contamination sources to be sewage and cow feces. If the unknown partition was interpreted as a contaminating source, an additional 10 and 32 communities were contaminated according to the LB-MST and DM-MST analyses, respectively. Thus, the number of contaminated samples and the identified contaminating sources differed substantially between the WB-MST setup and setups that included background information.

To compare the performance of the three MST setups to the corresponding FIB abundance data, we were interested in inferring correlations between obtained contaminations and FIB scores. A multivariate hierarchical GLMM was fitted to the contamination proportion data and FIB abundance. The model included contamination results from the three MST setups as different response variables and sample location as a grouping variable. We interpreted contamination from an unknown source in the DM-MST and LB-MST analyses as a contaminating source because it represents a deviation from the normal background composition of the sink assemblage. The contaminations obtained in the WB-MST analysis resulted in negative estimated correlations with Enterococci and Coliform abundance (**Table 2**), although the zero was included within the body of the 95% credible interval (CI). Contaminations predicted through the DM-MST and LB-MST analyses were positively correlated with Coliform abundance, with the zero outside the body of the 95% CI. Contaminations predicted through both models were also positively correlated with *Enterococci* abundance, but with the zero within the 95% CI. Interestingly, only contaminations obtained from the LB-MST analysis were strongly correlated with *E. coli* abundance; results from the other two analyses only showed a weak non-significant correlation with *E. coli* abundance. Results from the LB-MST analysis mirrored the results of traditional fecal indicators, i.e., significantly correlated with *E. coli* and Coliform abundance and, to a lesser extent, *Enterococci* abundance. Contaminations obtained in DM-MST and LB-MST analyses yielded strong positive correlations, while a negative estimated mean correlation of contaminations between WB-MST and DM-MST analyses and between WB-MST and LB-MST analyses were obtained, although the zero was within the range of the 95% most credible values.

**Table 2:** Point estimates of correlations between fecal indicators and the proportion of contaminations obtained in the MST analysis of raw water samples.

Response 1	Response 2	Mean	Median	Std	2.5% CI	97.5% CI
WB-MST	DM-MST	-0.237	-0.228	0.187	-0.621	0.132
	LB-MST	-0.073	-0.066	0.192	-0.490	0.293
	*E. coli*	0.093	0.099	0.210	-0.356	0.482
	Enterococci	-0.339	-0.343	0.195	-0.702	0.046
	Coliforms	-0.174	-0.162	0.186	-0.572	0.180
DM-MST	LB-MST	0.763	0.762	0.076	0.621	0.905
	*E. coli*	0.050	0.047	0.102	-0.151	0.256
	Enterococci	0.158	0.154	0.109	-0.059	0.372
	Coliforms	0.400	0.398	0.082	0.239	0.560
LB-MST	*E. coli*	0.260	0.257	0.096	0.071	0.451
	Enterococci	0.093	0.093	0.111	-0.115	0.312
	Coliforms	0.398	0.393	0.080	0.239	0.554
*E. coli*	Enterococci	0.736	0.741	0.069	0.584	0.855
	Coliforms	0.540	0.541	0.082	0.372	0.691
Enterococci	Coliforms	0.536	0.539	0.089	0.339	0.693

*The three MST library setups are: without background (WB-MST), with a local background (LB-MST), and background selected through clustering analysis (DM-MST). Mean, Median, Std, 2.5 and 97.5% Credible Intervals (CI) refer to respective posterior point estimates.*

The three MST setups were further tested by comparing the obtained contaminations in samples with good water quality (FIB levels below 25 MPN/100ml for *E. coli* and 100 MPN/100ml for Coliforms). Both the DM-MST and LB-MST analyses showed average proportions of contamination that were 100-fold lower than what was obtained through the WB-MST analysis (**Table 3**, *P* < 2.2e-16 for both comparisons). A similar trend was noted for water samples with slightly lower quality scores, but both WB-MST and DM-MST contamination results were 10-fold lower than what was obtained through LB-MST (*P* = 1.709e-13 and *P* = 1.596e-15, respectively). The average contamination scores increased as water quality deteriorated for both DM-MST and LB-MST, while an opposite trend was observed for WB-MST.

**Table 3:** The estimated proportions of contamination (standard deviation within parentheses) from the without background (WB-MST), with local background (LB-MST), and with DM-selected background (DM-MST) models.

MST setup	Good water quality	Unknown source added	Number of samples	Mean proportion classified as contamination
WB-MST	Yes	No	110	0.38 (0.17)
LB-MST	Yes	Yes	110	0.0032 (0.0064)
DM-MST	Yes	Yes	110	0.0057 (0.0098)
WB-MST	No	No	45	0.303 (0.176)
LB-MST	No	Yes	45	0.035 (0.069)
DM-MST	No	Yes	45	0.045 (0.077)

*Communities were divided into groups according to FIB threshold levels, with potential background water sample characterized by E. coli <25 MPN/100 ml and coliforms <100 MPN/100ml. The column ‘Unknown’ indicates whether the proportions grouped under unknown sources were merged with the fecal source proportions in DM-MST and LB-MST setups.*

### Prediction Accuracy Is Higher in Models That Include Background Assemblages

An important aspect of signature-based MST analysis is the accuracy of the obtained contaminating signals, as low prediction accuracy suggests a higher likelihood for false positives in terms of both inferred proportion and source. We evaluated signal quality generated by the three MST setups by assessing the OTU content of each contamination signal. To do so, we investigated the inferred posterior probability of all OTUs to originate from the respective source, estimated by the SourceTracker software, by counting how many OTUs that obtained an source origin probability greater or equal to 0.7: these are called source accurate OTUs. The average proportions of source accurate OTUs observed for the WB-MST, DM-MST, and LB-MST analyses were 0.046 (0.069), 0.200 (0.202), and 0.197 (0.195), respectively, with standard deviations shown in parentheses. A generalized linear model was fitted to the data using a log link function, with site, sample within the site, and source of the contamination set as the grouping variables. The effect of WB-MST on the proportion of accurate OTUs was set as the baseline, after which the effects of the alternative MST setups were estimated. The results show that both DM-MST and LB-MST positively influence the proportion of accurate OTUs relative to the WB-MST analysis (β_1_ = 1.34; 95% CI: 1.28, 1.39 for DM-MST and β_2_ = 1.30; 95% CI: 1.24, 1.35 for LB-MST). In addition, the narrow 95% CIs suggest high precision in the estimates and the zero (i.e., baseline effect) was well outside the 95% CIs. This implies that going from the WB-MST setup to a DM-MST setup (without changing any other predictors) would increase the expected proportion of accurate OTUs almost fourfold (i.e., e^β1^ = e^1.34^ = 3.82).

### The Analysis of a Real-Life Contamination Event Demonstrates How the Inclusion of Background Communities Impacts the Predicted Source of Pollution

To further compare the performances of the three MST setups, a real contamination scenario was analyzed in depth. In April 2014, raised FIB values at the inlet of the water treatment plant in Östersund indicated a fecal contamination event. Levels of *E. coli* and Coliforms were 53 and 147 MPN/100ml, respectively, which are markedly higher than the usual levels at this location (**Table 1**). Sewage was simultaneously detected at a nearby marina, and believed to be the source of the contamination. An inlet sample was analyzed with all three MST approaches to detect the contaminating sources. All three MST setups detected sewage as a contaminating source, although the WB-MST analysis reported a higher proportion of the dog fecal source (**Table 4**).

**Table 4:** Proportions of contamination, by source, estimated by the three MST setups: without background (WB-MST); with a local background (LB-MST); and with a background selected through clustering analysis (DM-MST).

Source	MST setup	Proportion (%)	Std	No of OTUs (*n*)	No of Accurate OTUs (*n*)
Background	WB-MST	-	-	-	-
Background	LB-MST	98.6	0.016	586	537
Background	DM-MST	97.5	0.031	561	488
Dog	WB-MST	63.9	0.201	264	41
Dog	LB-MST	0	0	–	–
Dog	DM-MST	0	0	–	–
Horse	WB-MST	0	0	–	–
Horse	LB-MST	0.124	0.015	37	2
Horse	DM-MST	0.267	0.007	46	8
Sewage	WB-MST	10.4	0.069	309	39
Sewage	LB-MST	1.21	0.023	289	130
Sewage	DM-MST	2.14	0.021	309	140
Unknown	WB-MST	25.7	0.221	420	210
Unknown	LB-MST	<0.10	0.006	201	8
Unknown	DM-MST	<0.10	0.008	199	13

**FIGURE 3:**
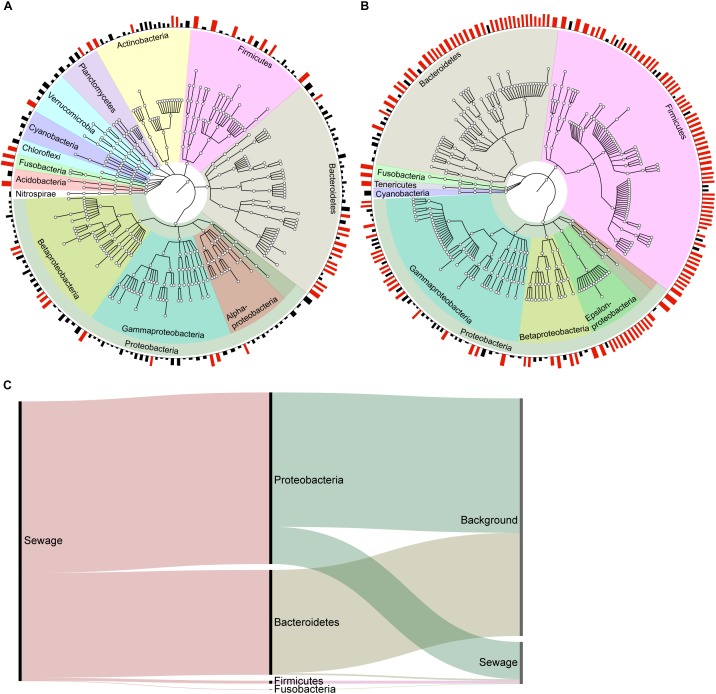
Visualization of the dog signal from the MST analysis without background representation in the library **(A)**, and of the sewage signal from the MST analysis with background representation selected through clustering analysis **(B)**. The plot visualizes the taxonomy of each detected OTU at the phylum level, and classes are visualized within the *Proteobacteria* phylum. The bars along the edge of the circle represent the posterior probability that the OTU belongs to the source, with probabilities ≥0.70 indicated by red bars. OTUs with source probabilities <0.10 were removed to facilitate visualization. **(C)** An alluvial diagram reveals the taxonomic structure of the sewage signal in the WB-MST analysis, as well as how this signal is divided in the DM-MST analysis. The height of the bars reflects the number of reads in each group.

Microbial source tracking analyses are favored because they can provide the taxonomic composition of each obtained signal, the number of OTUs within the signal, as well as the amount of source accurate OTUs. The OTUs in the dog signal obtained by WB-MST analysis represented a variety of phyla, such as Proteobacteria, Bacteroidetes, Firmicutes, Actinobacteria, and Planctomycetes (**Figure 3A**). The same set of phyla was represented in the unknown signal, while the sewage signal mainly comprising OTUs assigned to Proteobacteria, Bacteroidetes, and Firmicutes (**Supplementary Figure 7**). Many of the sewage source accurate OTUs belonged to the genus *Arcobacter*, within the class Epsilonproteobacteria. The observed proportion of source accurate OTUs supporting dog, sewage, and unknown signals were 0.16, 0.13, and 0.50, respectively (**Table 4**). This suggests that dog fecal and sewage signals mainly included OTUs with low source assignment probabilities (i.e., shared between sources).

We chose to only visualize the results obtained using DM-MST analysis because the DM-MST and LB-MST setups produced strikingly similar results. The OTUs identified in the sewage signal using DM-MST analysis represented the same set of phyla obtained through the model without background (**Figure 3B**). More specifically, the signal consisted of *Acinetobacter* (Gammaproteobacteria), *Arcobacter* (Epsilonproteobacteria), and *Bacteroides* (Bacteroidetes) genera as well as the Lachnospiraceae and Ruminococcaceae families within the Firmicutes phylum, all of which have been identified from freshwater contaminated with raw sewage (Newton et al., 2013). The observed proportions of source accurate OTUs were greater when the DM-MST or LB-MST setups were used than when the WB-MST setup was used (0.45 for both compared to 0.13) (**Table 4**). The background source signal from the DM-MST analysis consisted of a large set of OTUs in which all major phyla were represented (**Supplementary Figure 8A**), and both the LB-MST and DM-MST analyses were characterized by high proportions of source accurate OTUs (0.92 and 0.87, respectively). The horse signal included only a few OTUs (**Table 4** and **Supplementary Figure 8B**), with a low proportion of source accurate OTUs (0.05 and 0.17 for LB-MST and DM-MST, respectively).

We also investigated the reads underpinning the obtained signals to compare the performances of the MST setups. Approximately 85% of the WB-MST reads in the sewage signal supported the background signal obtained in DM-MST analysis and were assigned to Proteobacteria and Bacteroidetes phyla (**Figure 3C**). This implies that models that do not account for OTU overlap (i.e., between the background and sewage) run the risk of overestimating source proportions. Unsurprisingly, almost all of the reads underpinning dog and unknown source signals in the WB-MST analysis were suggested to originate from the background source in DM-MST analysis (**Supplementary Figure 9**). However, some reads assigned to the dog and unknown signals in the WB-MST analysis supported the sewage signal in the DM-MST analysis. In fact, approximately 74% of the reads in the sewage signal obtained from the DM-MST analysis were also included in the WB-MST sewage signal, whereas 6 and 19% of the reads assigned to sewage in the DM-MST analysis, most of which belonged to Firmicutes phylum, had been grouped in the dog and unknown sources, respectively, during WB-MST. This implies that information about contamination may be assigned to wrong sources if the overlap between raw water and fecal communities is not accounted for. The reads forming the basis for the background signal in DM-MST was distributed across all sources obtained in the WB-MST analysis: 71% to dog, mainly Actinobacteria and Proteobacteria; 19% to unknown via all major phyla with the exception of Firmicutes; and 10% to sewage via Bacteroidetes and Proteobacteria.

## Discussion

Accurately tracking the source of water contamination is key to fully understanding potential risks and enabling remediation. Advances in high-throughput sequencing have provided researchers the opportunity to utilize signature-based MST methods when deciphering traces of contamination from point sources (Newton et al., 2013; Ahmed et al., 2015; Henry et al., 2016). The success of sequence-based MST analysis depends on the degree of OTU overlap between sources, as specified in a source tracking library. However, only a few studies provide guidelines for how to model OTU overlap and how a source tracking library should be defined to incorporate natural variation in the raw water bacterial community. To address this knowledge gap, three alternatives for library setup were evaluated. The obtained results revealed two major benefits for incorporating background information into the MST library: (i) correlations between predicted contamination and FIB scores improved; and (ii) the probability of assigning an OTU to the correct contamination source increased, on average, fourfold.

The LB-MST results for raw water samples collected from six Swedish sites were positively correlated with the traditional culture-based FIB measures. DM-MST and LB-MST analyses yielded highly similar estimated proportions of contamination, and both were negatively correlated to contaminations estimated by the WB-MST analysis, especially in regards to *Enterococci* abundance. Ahmed et al. (2015) analyzed fecal pollution events in Australian waters without including any background communities in the MST library. They did not find any positive associations between FIB (*E. coli* and *Enterococcus*), host-associated molecular markers, and 16S rRNA amplicon data, but found a statistically significant negative association between estimated sewage proportion and *E. coli* levels. On the other hand, Newton et al. (2013) did find significant positive correlations between *Enterococci* and *E. coli* levels and the obtained magnitudes of sewer and fecal signatures in samples from urban rivers, storm water, and a harbor in Milwaukee, as well as Lake Michigan. These signatures were based on a selection of pre-defined genera and families, associated to human feces. However, as no background information was included in the MST library, non-significant, slightly negative associations between FIB levels and the human fecal signals obtained through SourceTracker software were reported. On the other hand, an analysis of recreational coastal sites in Victoria, Australia, (Henry et al., 2016) that included local background communities in the MST library found the detected proportions of sewage and graywater to be positively associated with *Enterococci* levels. These results agree with the findings presented for MST approaches that include source datasets. This highlights the importance of including bacterial overlap between communities in the library, which is particularly crucial for sources that share a large set of OTUs, such as chronically polluted environmental waters and raw sewage. Interestingly, Dubinsky et al. (2016) included blank samples in the MST library to control for OTUs that stem from the laboratory environment. Such a setup is likely to further improve the signal-to-noise ratio and correlation to traditional fecal indicator levels.

In this study, we suggest characterizing signals obtained from MST, which may represent contaminating sources or background variation in the taxonomic composition of a watershed, based on the proportion of source accurate OTUs. On average, the proportion of source accurate OTUs were significantly higher in models that included background communities, i.e., LB-MST and DM-MST as compared to WB-MST. In other words, source predictions based on OTUs included a higher degree of confidence when the MST library included background communities. Few signature-based MST analyses have used OTUs underpinning the predictions to draw further conclusions about the contaminating source. Newton et al. (2013) used the source probabilities of OTUs to derive a human fecal signature and screened sink samples of urban rivers in Milwaukee, a harbor, and Lake Michigan for this signature. The abundance of this signature in the sink samples correlated well with events of combined sewage overflows, suggesting that using signatures based on the source probabilities might be an interesting alternative to evaluating contamination source predictions inferred by the MST algorithm.

The information provided by the set of OTUs in a contaminating signal opens up corridors for future research. The presented results suggest that proportions of source accurate OTUs can be used to dismiss false positives when few source accurate OTUs are correctly identified; for example, the horse contamination source identified in the case study was based on few source accurate OTUs, and the proportion of source accurate OTUs was much lower than what was observed for raw sewage by DM-MST and LB-MST. Future research could compare this approach for reducing false-positives with a method based on relative standard deviation put forward by Henry et al. (2016). Another interesting possibility is to utilize the OTUs and their phylogenetic relationships to develop markers for specific contaminating sources. Such an approach could specifically target fecal-associated anaerobes, which are typically more abundant than the classic cultivable indicators (McLellan and Eren, 2014). An ideal target for marker development would be phylogenetically distinct from other fecal-derived targets, thus avoiding cross-reactions with other groups, and should be accurately assigned to the correct group by MST analysis. An example of one such target could be the OTUs assigned to Epsilonproteobacteria, which was associated with the sewage signal in the case study. The Epsilonproteobacteria class contains some well-known fecal associated pathogens, of which Campylobacter and Helicobacter may be the most relevant in terms of human health risks (Kärenlampi et al., 2007; Khalifa et al., 2010; Man, 2011).

All three MST setups detected sewage contamination in the case application. The WB-MST analysis estimated a large proportion of dog fecal contamination, which was absent from the results of MST setups that included background communities in the library. Most of the reads supporting the dog signal in the WB-MST analysis supported the background source in the DM-MST analysis, although some reads did support a sewage signal. Thus, the absence of a defined OTU overlap between dog feces and raw water most likely caused the overestimation of dog contamination in the WB-MST analysis. The dog communities (i.e., the average distance to its centroid) showed the highest dispersion of all environments in the library, which may explain the large number of OTUs shared between the raw water and dog sources. Although we cannot rule out the possibility of chronic pollution events caused by dog feces at this watershed, it nevertheless seems an unlikely scenario. This is because other MST studies of contaminated environmental water have been identified wild bird (Dubinsky et al., 2012), cattle (Hagedorn et al., 1999), and human feces (Newton et al., 2013) as the most common contaminating sources, with dog fecal pollution less frequent (Whitlock et al., 2002). Taken together, these results suggest that the dog fecal source detected in the WB-MST analysis is a false-positive and, by accounting for background OTU composition, erroneous detections can be circumvented. To further strengthen this claim, MST setups that included background assemblages yielded very few reads with an unknown source origin, indicating a complete, representative library for assessing water contamination.

Another cause of concern is the degree of OTUs shared between fecal sources in the library. A high degree of shared OTUs will make it difficult to distinguish two environments through MST analysis, particularly if the contaminating signal is weak with respect to low source probability of OTUs and/or a predicted proportion of contaminating source that is close to the noise level. For example, in the presented analyses, dog and sewage environments shared a relatively large number of OTUs. Such overlap could potentially cause biases in the contamination source estimation. However, as almost all of the reads in the dog signal from the WB-MST setup were assigned to the background signal during DM-MST analysis, we believe that overlap between the studied water environments and potential contaminants is the biggest concern, and should be addressed via the inclusion of representative water communities in the MST library.

Our results suggest that incorporating representative water communities into MST analysis improves both accuracy, in terms of assignment to the correct source, and estimated proportions of contamination. The presented results suggest that using either communities sampled at the same location (LB-MST) or selected from a set of representative communities (DM-MST) serve as equally good alternatives, as shown in the real case study. It seems reasonable to suggest that using local communities, if possible, would be preferable as bacterial community composition is influenced by geographic distance and time (Yannarell and Triplett, 2005; Fierer et al., 2007; Hanson et al., 2012). However, in typical MST cases, no sampling of the local environment has been performed and, as such, no local reference assemblages are available. A convenient alternative, which is based on selecting representative communities via cluster analysis, relies on the ever increasing amount of sequence bacterial communities in public databases, such as the MG-RAST server (Glass et al., 2010): this increase will enhance the performance of the MST without any local sampled assemblages available. The cluster based DM-MST would be able to select assemblages of similar character as the sink sample, and if strong spatiotemporal variation exists, nearby sampled communities, both in time and in geographic location, would be selected as a background. Therefore, this MST setup (or a combination of LB-MST and DM-MST) is perhaps best suited if strong spatiotemporal effects are shaping the community composition. To further evaluate whether using a local or selected representative communities is better, the impact of spatial and temporal effects on MST performance need to be studied in more detail, by performing large scale longitudinal sampling effort.

## Conclusion

The inclusion of background assemblages in the library greatly benefits MST analyses. The results from models that include background water data were significantly and positively correlated to measured FIB levels, which suggest that these analyses can be used to identify human health risks from a water sample. Moreover, these models were characterized by improved accuracy in identifying the source of a contaminant when compared to models that did not include any background information. We believe that investigating the proportion of OTUs in a signal with high source accuracy provides a solid foundation to further decipher and compare contaminating signals.

Multiple-signature MST approaches offer various advantages over traditional culture or molecular methods, which can only detect a small portion of contaminating microbes and offer limited information about contamination diversity and raw water composition. High-throughput sequencing techniques bypass the need for isolation or cultivation of microorganisms, and provide insight into the environmental metagenome, which can be used to improve MST accuracy. As shown in this study, accounting for the bacterial community overlap between background raw water and contaminating sources greatly improves the accuracy at which an analyzed contaminant is assigned to the correct source. This tool can be used to pinpoint effective measures for minimizing the spread of pathogenic and antibiotic resistant bacteria into essential freshwater resources.

## Data Availability

Supplementary data related to this article can be accessed at the Short Read Archive (https://www.ncbi.nlm.nih.gov/sra), project reference SRP159537. Filtered OTU tables are provided in **Supplementary Material 1**.

## Author Contributions

SB, AM, EB, KJ, and RD collected the data. MH and JA processed the data after sequencing. MH and JA conducted data analyses. MH and JA wrote the manuscript. SB, AM, PL, KJ, RD, PS, AS, and MF contributed to writing the manuscript. KJ, RD, and MF organized and supervised the data collection. KJ, RD, PS, AS, MF, and JA supervised the project.

## Conflict of Interest Statement

The authors declare that the research was conducted in the absence of any commercial or financial relationships that could be construed as a potential conflict of interest.
