# Supplementary material for: Accounting for Bacterial Overlap Between Raw Water Communities and Contaminating Sources Improves the Accuracy of Signature-Based Microbial Source Tracking
Source: Front Microbiol. 2018 Oct 2;9:2364. doi: 10.3389/fmicb.2018.02364 (PMC6190859; doi:10.3389/fmicb.2018.02364)
Supplement: Supplementary file 2 [file Data_Sheet_2.docx]

Supplementary Material

# **Accounting for bacterial overlap between raw water communities and contaminating sources improves the accuracy of signature-based microbial source tracking**

**Moa Hägglund^1^, Stina Bäckman^1^, Anna Macellaro^1^, Petter Lindgren^1^, Emmy Borgmästars^2^, Karin Jacobsson^2^, Rikard Dryselius^2^, Per Stenberg^1,3,4^, Andreas Sjödin^1,4^, Mats Forsman^1,5^, Jon Ahlinder^1*^**

^1^ Division of CBRN Security and Defence, FOI, Swedish Defence Research Agency, SE-906 21, Umeå, Sweden

^2^ National Food Agency, SE-751 26, Uppsala, Sweden

^3^ Department of Molecular Biology, Umeå University, SE-901 87 Umeå, Sweden

^4^ Department of Chemistry, Computational Life Science Cluster (CLiC), Umeå University, SE-901 87 Umeå, Sweden

^5^ Department of Clinical Microbiology, Umeå University, SE-901 87 Umeå, Sweden

*** Correspondence:**

Jon Ahlinder

E-mail: [jon.ahlinder@foi.se](mailto:jon.ahlinder@foi.se)

**Supplementary Tables**

**Supplementary Table 1.** Statistics for the sequencing of fecal samples. Cows contributing feces were over 24 months old while calves were all younger than two months. Standard deviation is shown in parentheses.

| Sample group | Number of samples in group | Median number of reads | Minimum number of reads | Maximum number of reads | Phylogenetic diversity | Geographical origin |
| --- | --- | --- | --- | --- | --- | --- |
| Calf feces | 10 | 173,605 | 107,285 | 194,873 | 52.9 (15.2) | Uppsala |
| Cow feces | 25 | 53,800 | 15,341 | 129,589 | 72.2 (7.6) | Borlänge, Falköping, Borås, Säter, Uppsala, Väderstad |
| Dog feces | 25 | 77,036 | 29,389 | 122,614 | 44.6 (32.7) | Borlänge, Falun, Halmstad, Mariestad, Uppsala, Tibro, Tierp, Väderstad |
| Domestic bird feces | 25 | 50,871 | 15,216 | 102,193 | 42.2 (15.3) | Falköping, Halmstad, Borås, Tierp, Uppsala |
| Horse feces | 14 | 87,431 | 15,553 | 135,028 | 80.5 (20.3) | Borlänge, Falun, Uppsala |
| Pig feces | 25 | 78,495 | 51,145 | 187,875 | 84.0 (17.0) | Halmstad, Lidköping, Borås, Skövde, Uppsala |
| Sewage inlet | 10 | 49,527 | 36,193 | 119,993 | 182.6 (21.5) | Bålsta, Enköping. Knutby,  Gåvsta,  Länna,  Storvreta, Umeå, Uppsala |
| Sheep feces | 10 | 96,822 | 83,173 | 433,914 | 105.2 (16.5) | Uppsala |
| Wild bird feces | 14 | 91,051.5 | 52,822 | 193,533 | 38.8 (6.3) | Hudiksvall, Kalmar, Uppsala |

#

# **Supplementary Figures**


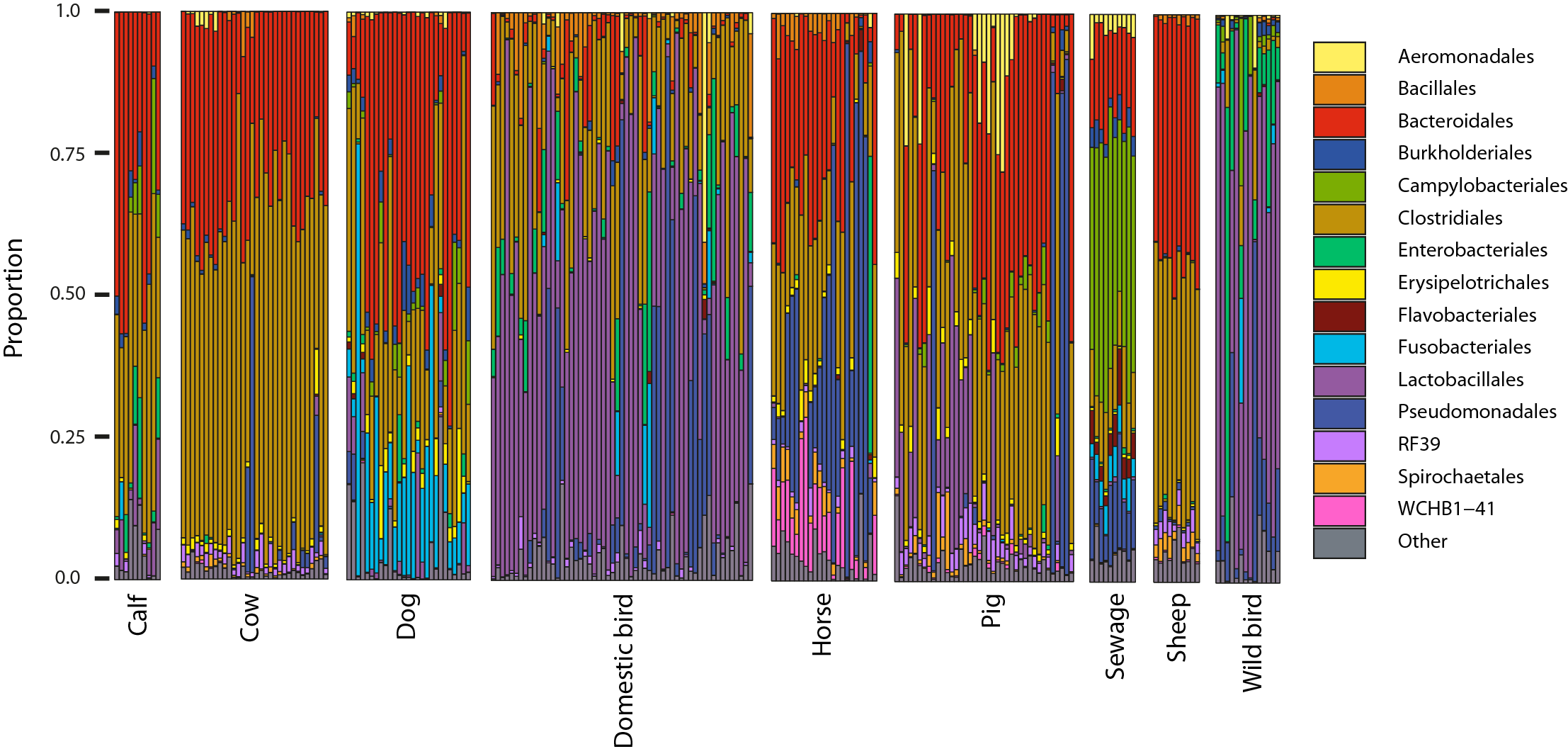


**Supplementary Figure 1.** Taxonomic composition, at the order level, of the included fecal and sewage samples sorted by source type.


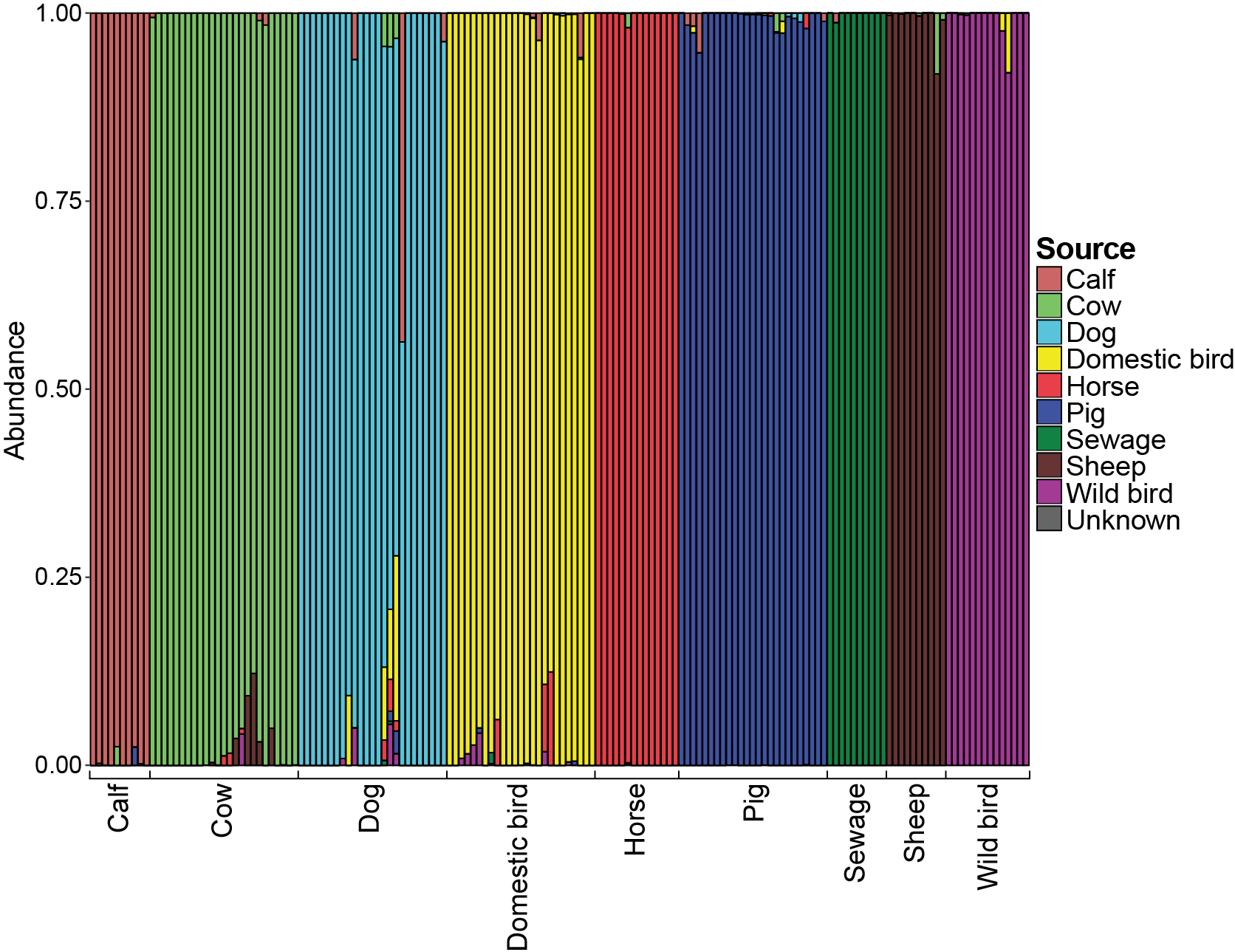


**Supplementary Figure 2.** Result of the leave-one-out cross-validation analysis of the contaminating sources included in the MST library.


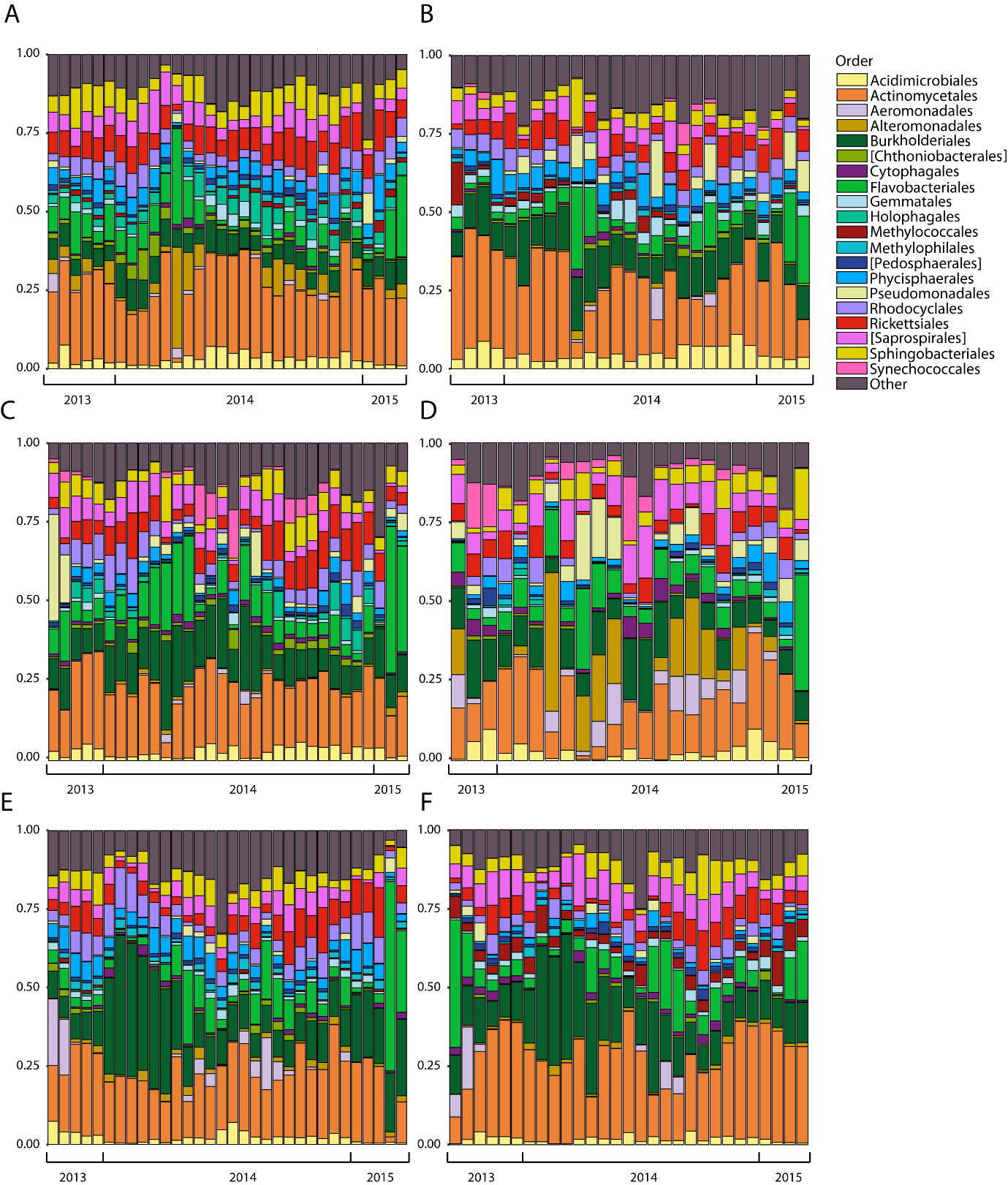


**Supplementary Figure 3.** Temporal taxonomic composition, at the order level, of the sampled raw water from drinking water treatment plant inlets. The locations of the plants are as follows: **A** Stockholm; **B** Östersund; **C** Trollhättan; **D** Motala; **E** Borås; and **F** Härnösand.


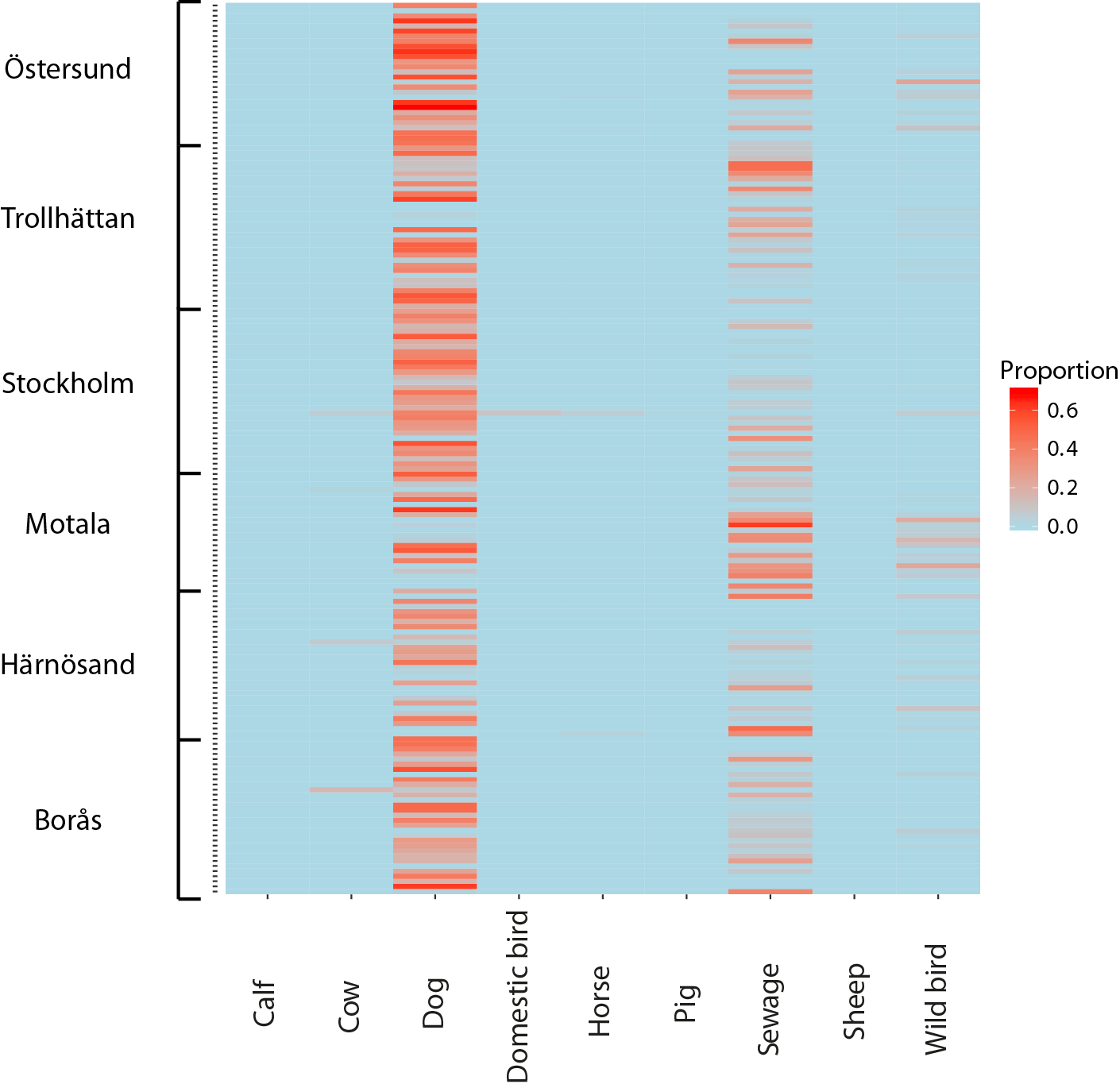


**Supplementary Figure 4.** Heatmap of the proportions of contaminations in each raw water community estimated by the WB-MST library setup, with locations displayed on the y-axis and contaminating sources displayed on the x-axis.


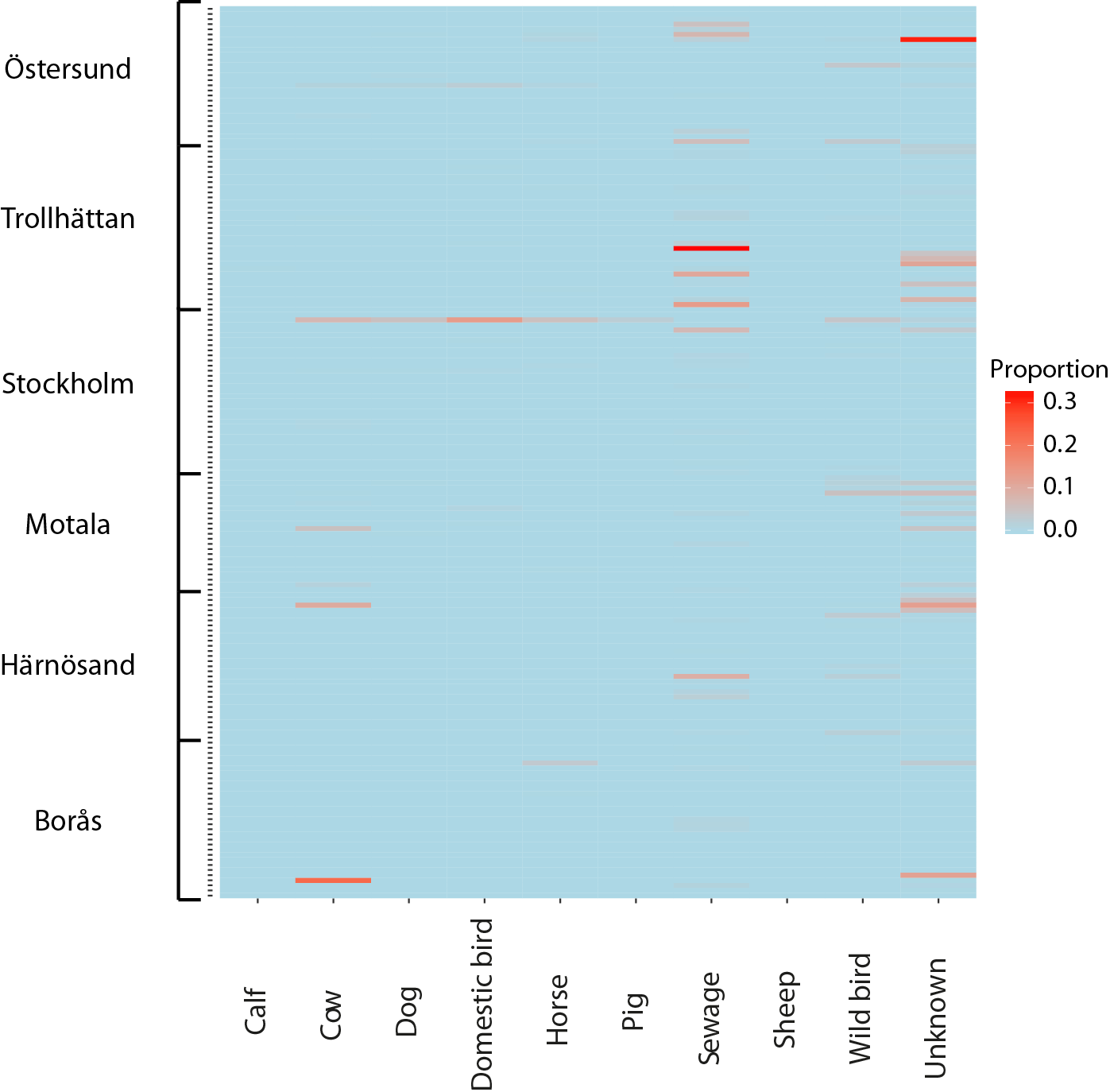


**Supplementary Figure 5.** Heatmap of the proportions of contaminations in each raw water community estimated by the LB-MST library setup, with locations displayed on the y-axis and contaminating sources displayed on the x-axis.


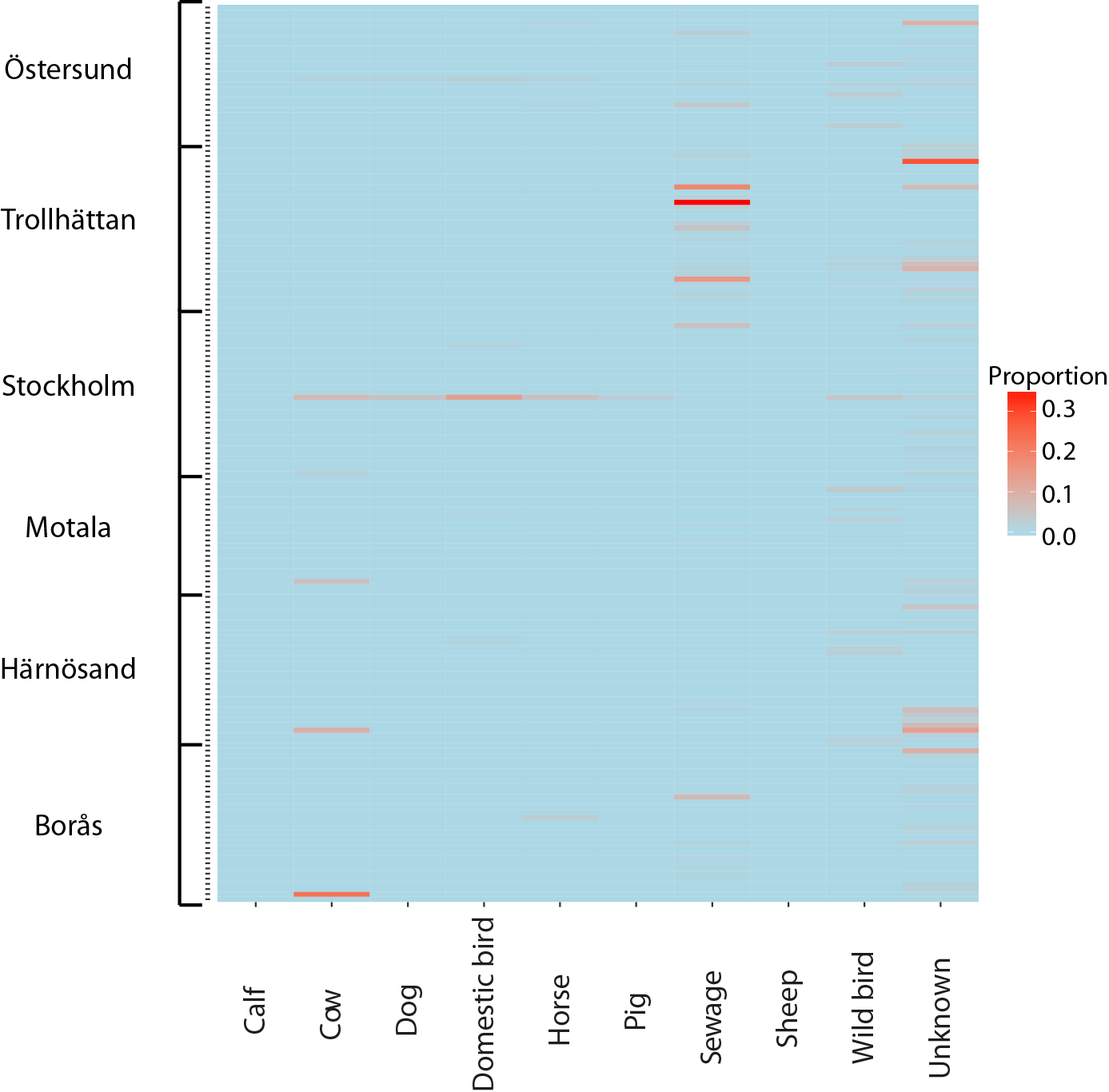


**Supplementary Figure 6.** Heatmap of the proportions of contaminations in each raw water community estimated by the DM-MST library setup, with locations displayed on the y-axis and contaminating sources displayed on the x-axis.


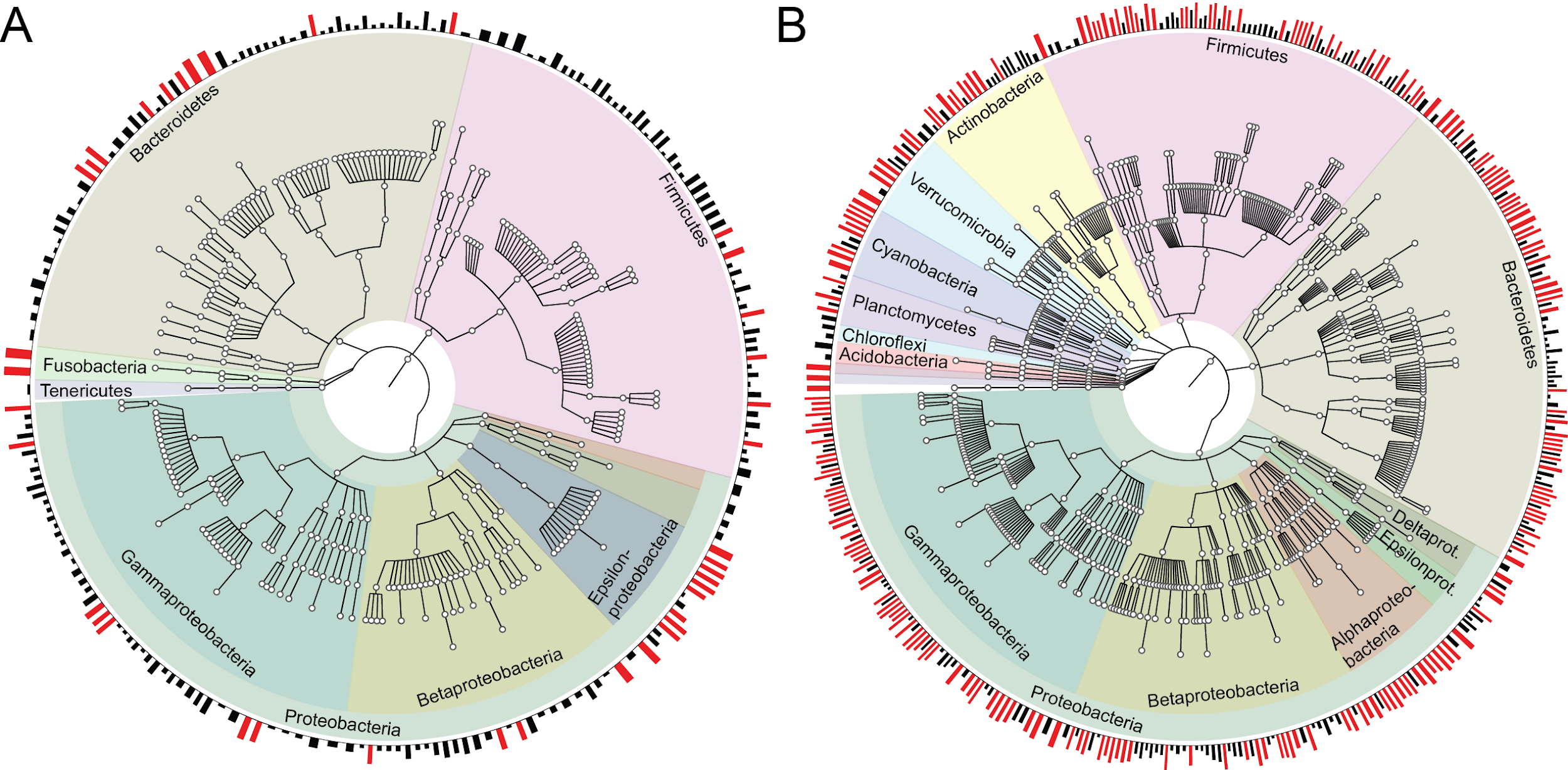


**Supplementary Figure 7.** Visualization of the sewage (**A**) and unknown (**B**) signals from the MST without background communities included in the library. The plot illustrates the taxonomy, displayed at the phylum level, of each detected OTU. Classes are visualized within the Proteobacteria phylum. The bars along the edge of the circle represent the posterior probability that an OTU belongs to the source, with probabilities ≥0.70 is indicated with red bars. OTUs with source probability <0.10 were removed to facilitate visualization.


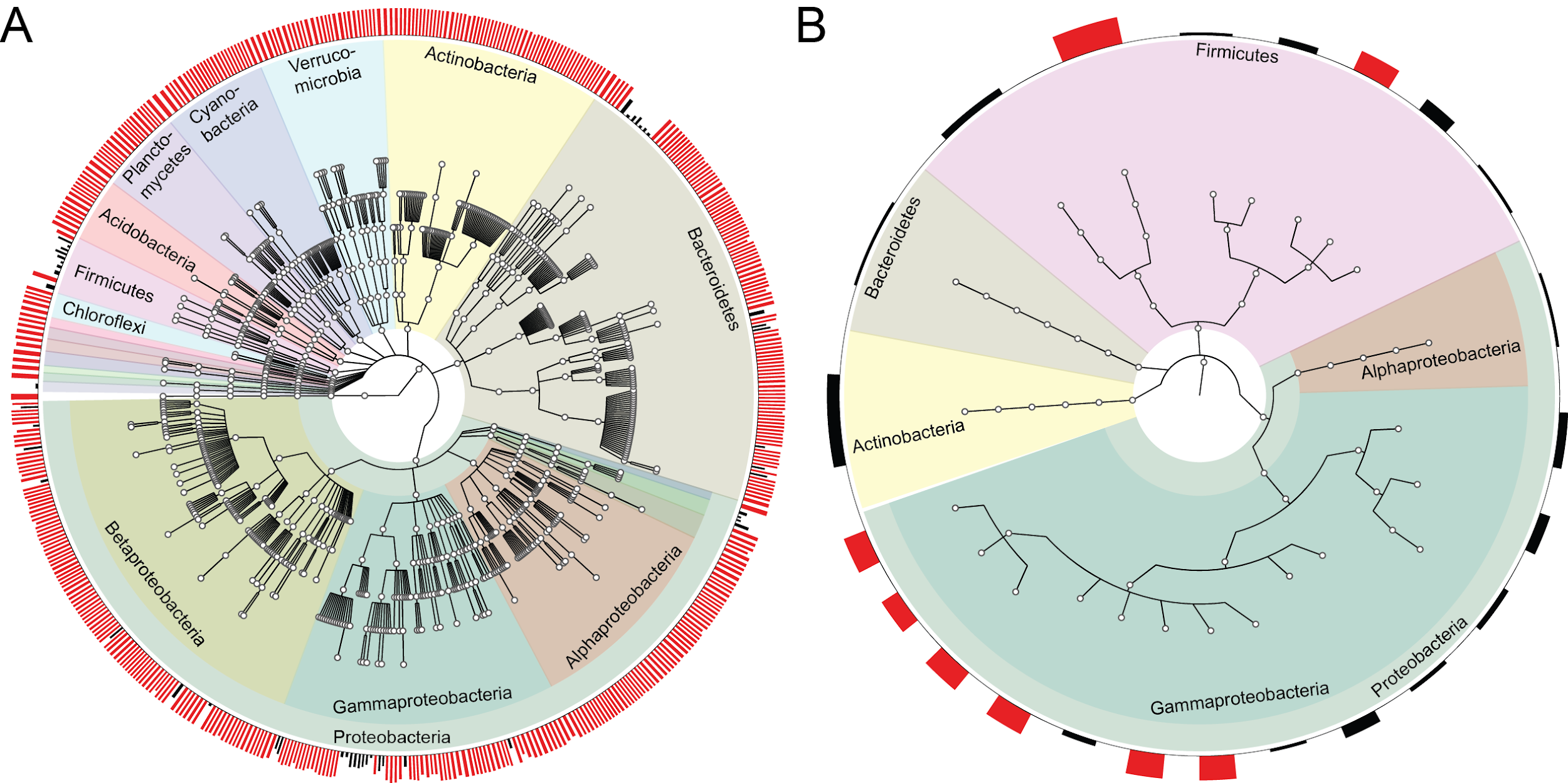


**Supplementary Figure 8.** Visualization of the background (**A**) and horse (**B**) signals in the MST analysis that included background communities selected through clustering analysis. The plot illustrates the taxonomy, displayed at the phylum level, of each detected OTU. Classes are visualized within the Proteobacteria phylum. The bars along the edge of the circle represent the posterior probability that an OTU belongs to the source, with probabilities ≥0.70 indicated with red bars. OTUs with source probability <0.10 were removed to facilitate visualization.


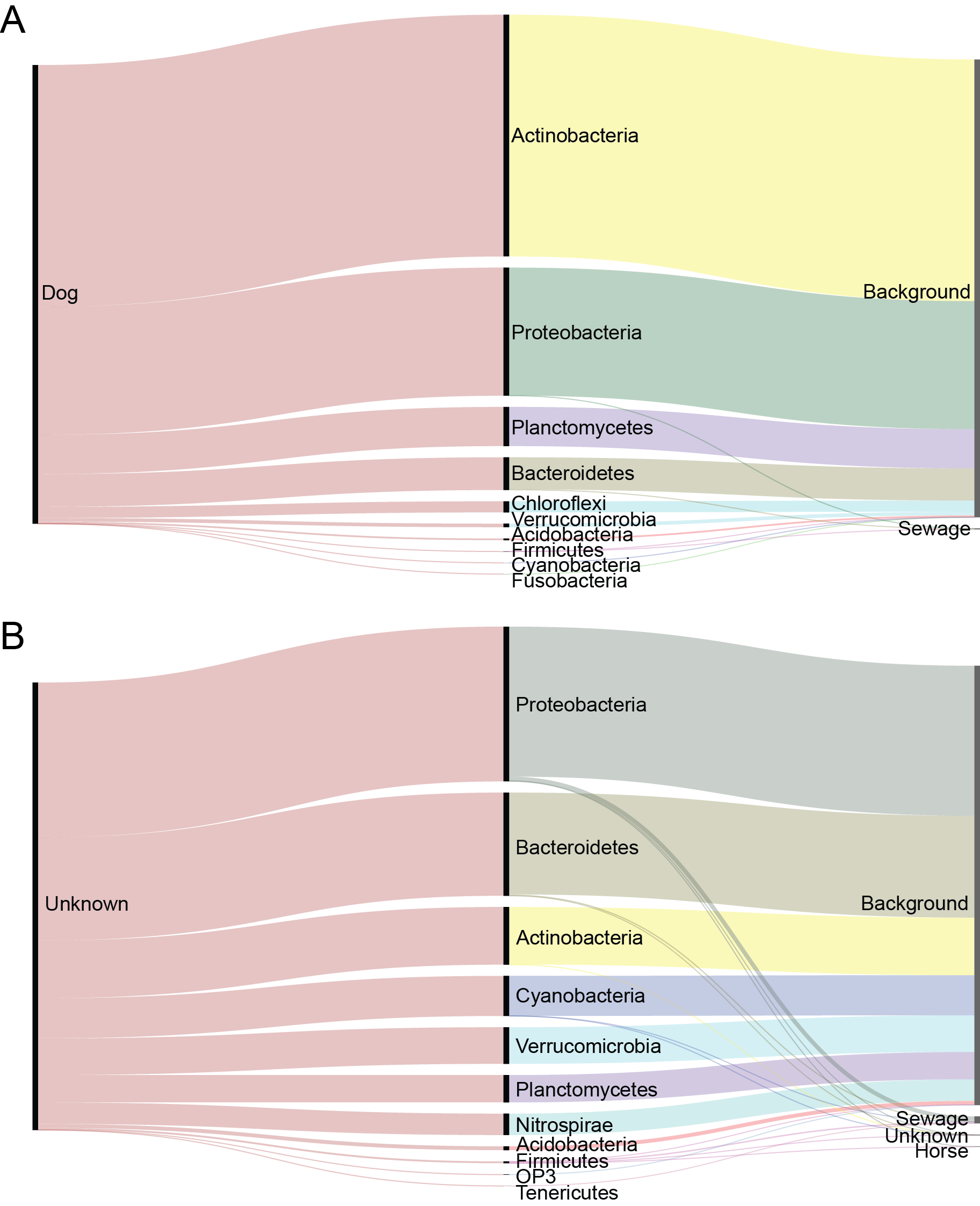


**Supplementary Figure 9.** Alluvial diagrams reveal the taxonomic structure of the dog (**A**) and unknown (**B**) signals from the WB-MST analysis, and how this signal is divided in the DM-MST analysis (right side of diagram). The height of the bars reflects the number of reads in each group.
